# Identifying biomarkers for methotrexate efficacy in rheumatoid arthritis: a machine learning approach to whole-blood transcriptomic analysis

**DOI:** 10.1093/rheumatology/keag560

**Published:** 2026-10-08

**Authors:** Chuan Fu Yap, Nisha Nair, Suzanne M M Verstappen, Kimme Hyrich, Guillermo Barturen, María Rivas-Torrubia, Marta Gut, Ivo Gut, Marta E Alarcón Riquelme, Anne Barton, Darren Plant, RAMS Co-Investigators, Ade Adebajo, Khalid Ahmed, Atheer Al-Ansari, Roshan Amarasena, Marwan Bukhari, Margaret Callan, Easwaradhas G Chelliah, Hector Chinoy, Annie Cooper, Bhaskar Dasgupta, Martin Davis, James Galloway, Andrew Gough, Michael Green, Nicola Gullick, Jennifer Hamilton, Waji Hassan, Samantha Hider, Kimme Hyrich, Sanjeet Kamath, Susan Knight, Suzanne Lane, Martin Lee, Sarah Levy, Lizzy Macphie, Christopher Marguerie, Tarnya Marshall, Catherine Mathews, Frank McKenna, Sophia Naz, Mark Perry, Louise Pollard, Brian Quilty, Lindsay Robertson, Dipak Roy, Paul Sanders, Vadivelu Saravanan, David Scott, Gillian Smith, Richard Smith, Deborah Symmons, Lee-Suan Teh, Nick Viner

**Affiliations:** Centre for Genetics and Genomics Versus Arthritis, Centre for Musculoskeletal Research, The University of Manchester, Manchester, UK; Centre for Genetics and Genomics Versus Arthritis, Centre for Musculoskeletal Research, The University of Manchester, Manchester, UK; NIHR Manchester Biomedical Research Centre, Manchester University NHS Foundation Trust, Manchester Academic Health Science Centre, Manchester, UK; NIHR Manchester Biomedical Research Centre, Manchester University NHS Foundation Trust, Manchester Academic Health Science Centre, Manchester, UK; Centre for Epidemiology Versus Arthritis, Centre for Musculoskeletal Research, The University of Manchester, Manchester, UK; NIHR Manchester Biomedical Research Centre, Manchester University NHS Foundation Trust, Manchester Academic Health Science Centre, Manchester, UK; Centre for Epidemiology Versus Arthritis, Centre for Musculoskeletal Research, The University of Manchester, Manchester, UK; GENYO, Centre for Genomics and Oncological Research Pfizer, University of Granada, Andalusian Regional Government, PTS Granada, Granada, Spain; Department of Genetics, Faculty of Science, University of Granada, Granada, Spain; Bioinformatics Laboratory, Biotechnology Institute, Centro de Investigación Biomédica, PTS, Granada, Spain; GENYO, Centre for Genomics and Oncological Research Pfizer, University of Granada, Andalusian Regional Government, PTS Granada, Granada, Spain; Centro Nacional de Análisis Genómico (CNAG), Barcelona, Spain; Universitat de Barcelona (UB), Barcelona, Spain; Centro Nacional de Análisis Genómico (CNAG), Barcelona, Spain; Universitat de Barcelona (UB), Barcelona, Spain; GENYO, Centre for Genomics and Oncological Research Pfizer, University of Granada, Andalusian Regional Government, PTS Granada, Granada, Spain; Insitute for Environmental Medicine, Karolinska Institute, Stockholm, Sweden; Centre for Genetics and Genomics Versus Arthritis, Centre for Musculoskeletal Research, The University of Manchester, Manchester, UK; NIHR Manchester Biomedical Research Centre, Manchester University NHS Foundation Trust, Manchester Academic Health Science Centre, Manchester, UK; Centre for Genetics and Genomics Versus Arthritis, Centre for Musculoskeletal Research, The University of Manchester, Manchester, UK; NIHR Manchester Biomedical Research Centre, Manchester University NHS Foundation Trust, Manchester Academic Health Science Centre, Manchester, UK

**Keywords:** methotrexate, rheumatoid arthritis, machine learning, biomarker, DMARD

## Abstract

**Objectives:**

Methotrexate (MTX) is the first-line treatment for rheumatoid arthritis (RA), yet inadequate response is reported in 30–40% of patients. Predicting MTX response early could enable more personalized and effective treatment. This study aimed to identify biomarkers predictive of MTX response at 6 months through whole-blood transcriptomic signatures using machine learning.

**Methods:**

RNA sequencing data were generated in whole-blood samples taken from 100 MTX-naive RA patients at baseline (pre-treatment) and following 4 weeks post treatment with MTX. Machine learning models were trained to classify MTX response following 6 months of treatment using gradient boosted trees and interpreted using SHapley Additive exPlanations (SHAP) values to identify predictive genes.

**Results:**

Machine learning models trained on baseline and 4-weeks data achieved AUCs of 0.89 and 0.90, respectively. Stability of SHAP values showed that the baseline model was generally more stable, and therefore potentially more generalizable. Key predictive genes at baseline, which were downregulated in responders, included *CAV1*, *LCN12* and *GLB1L*.

**Conclusion:**

Blood-based gene expression profiling at baseline and after 4 weeks of MTX treatment can predict treatment response with high confidence revealing relevant gene pathways and candidate gene targets but requires independent validation. These findings highlight the potential for transcriptomic biomarkers to inform early treatment decision in RA, supporting precision medicine approaches.

*Rheumatology* key messages

- Not all patients with rheumatoid arthritis have an adequate response to methotrexate treatment.
- There is a need for biomarkers to predict whether patients will respond positively.
- A classifier based on whole-blood transcriptomics demonstrates potential for identifying biomarkers of drug response.

## Introduction

The treatment pathway for rheumatoid arthritis (RA) includes conventional synthetic disease modifying anti-rheumatic drugs (csDMARD) [1]. Methotrexate (MTX) is the cornerstone csDMARD that is received by 60–88% of treated patients [2]. Despite its broad use, ∼30–40% of RA patients on MTX do not experience satisfactory disease control [3]. If symptoms are not effectively controlled by MTX, patients may receive another csDMARD (e.g. sulfasalazine) or may be escalated to receive an advanced targeted therapy (e.g. biologics) [4]. Inception cohort data have shown that early and effective treatment within the first 6 months of symptom onset can influence outcome decades later [5]. As response to first medication can have long-lasting effects and because the cost of advanced targeted therapies is reducing, being able to predict which patients would benefit most from MTX as a first-line therapy would be a significant clinical advancement.

Several clinical factors, including age, sex seropositivity and disability scores have shown moderate success in predicting MTX efficacy [6]; with female sex, seropositive disease and higher health assessment questionnaire scores (HAQ), a measure of functional disability, correlating with a less favourable response [7]. However, clinical models alone are not sufficiently predictive to guide clinical management, and they do not capture information on relevant biological pathways [7].

Genetic and genomic factors have been explored as potential predictors of MTX efficacy identifying biomarkers in transcriptomic [8], proteomic [9] and metabolomics modalities [10]. However, many previous studies have limited biomarker data assessment to a single timepoint, missing the opportunity to assess on-treatment change in biomarker profiles.

The current study aims to utilize comprehensive whole-blood RNA sequencing data collected at baseline and following 4 weeks on MTX and apply machine learning to identify pre- and early treatment biomarker profiles that are predictive of MTX treatment response, assessed at 6 months on therapy.

## Methods

### Patient cohort

The patients in this study were already taking part in the Rheumatoid Arthritis Medication Study (RAMS, Manchester NHS Research Ethics Committee reference 08/H1008/25), a national multicentre, longitudinal observational study in the UK that recruited patients with RA who were about to commence MTX therapy for the first time [7], and all patients provided written consent. RAMS patients included in the current study had a diagnosis of RA according to the American College of Rheumatology 1987 revised criteria for the classification of RA [11], were of European ancestry, were about to receive treatment with MTX for their RA symptoms and had available whole-blood samples for RNA extraction, and clinical data to address the study aims. In total, 100 MTX-treated patients were selected for this study for bulk transcriptomics at baseline and at 4 weeks follow-up. Treatment response was assessed at 6 months after starting therapy using the European League Against Rheumatism (EULAR) criteria [12], where good responders were defined as patients with a change in disease activity score in 28 joints (DAS28-CRP) of >1.2 and a DAS28-CRP of <3.2 at 6 months, moderate responders were defined as patients with a change in DAS28-CRP of >0.6 and a DAS28-CRP of <1.2 at 6 months and non-responders were defined as patients with a change in DAS28-CRP of <0.6 at 6 months. Good and moderate responders were grouped together as ‘responders’ for analytical purposes.

### RNA sequencing

RNA-Seq libraries were generated as previously described [13]. Briefly, libraries were enriched using oligo-dT beads from 500 ng of total RNA using the TruSeq stranded mRNA Library Prep Kit. Double-stranded cDNA was adenylated 3' and ligated to indexed adaptors with unique molecular identifiers (Integrated DNA Technologies) before being amplified by 15 PCR cycles. Library quality was assessed using the Bioanalyzer DNA 7500 assay (Agilent Technologies, Santa Clara, CA, USA). Sequencing was performed on a NovaSeq 6000 (Illumina, San Diego, CA, USA) in paired-end mode with 2 × 151 bp read length. Image analysis, base calling and quality scoring were processed using RTA 3.4.4 analysis software, followed by the generation of FASTQ sequence files. Reads were aligned to the GENCODE v39 hg38 human reference genome using STAR [14] and gene expression levels were quantified using RSEM [15].

### Differential expression analysis

Genes with cumulative counts under 500 across samples and those expressed in <20% of samples were excluded. The glmmSeq R package [16] was used for differential gene expression analysis using a generalized linear mixed model with negative binomial distribution, including patient variability as a random effect. DESeq2 [17] was used to estimate dispersion parameters. Statistical models were adjusted for sex, baseline DAS28-CRP score and an interaction term was specified between sampling timepoint and treatment response. Sex was chosen as a covariate due to the female predominance of the disease being treated, and DAS28-CRP was included because preliminary cohort comparisons indicated a significant difference in baseline disease activity between responders and non-responders. The resulting model is:


$$y_{\mathrm{ij}}∼NB(\mu_{ij},\theta )$$

$$\text{log}(\mu_{ij})=\beta_{0}+\beta_{1}{\text{Sex}}_{i}+\beta_{2}{\text{BaselineDAS}28\text{CRP}}_{i}+\beta_{3}{\text{Response}}_{\mathrm{ij}}+\beta_{4}{\text{Timepoint}}_{\mathrm{ij}}+\beta_{5}({\text{Timepoint}}_{\mathrm{ij}}\times {\text{Response}}_{\mathrm{ij}})+u_{i}$$

Where *y_i_*_j_ is the gene expression for patient *i* at observation *j*; β_0_ is the intercept; β_1_ and β_2_ are fixed-effect coefficients for the covariates sex and baseline DAS28-CRP scores, respectively; β_3_, β_4_, and β_5_ are fixed-effect coefficients for response, timepoint and their interaction, respectively; and u_i_∼N(0,$\sigma_{u}^{2}$) is the random effect term for patient variability. Sex and baseline DAS28-CRP were included as covariates in the models to account for potential confounding. Baseline DAS28-CRP differed between responders and non-responders, while sex was included *a priori* as a clinically relevant variable in RA. A false discovery rate (FDR) below 0.05 and a fold-change exceeding 1.25 in either the interaction or treatment response identified genes as differentially expressed.

### Machine learning

DESeq2’s variance stabilizing transformation (VST) was applied for normalizing variance across genes prior to multivariate analyses. Features going into the model included normalized gene expression values, sex and baseline DAS28-CRP scores. Training of Gradient Boosted Trees Classifier models was carried out using the XGBoost library [18]. XGBoost was chosen due to its ability to handle class-imbalanced dataset with weights on classes, high suitability for tabular data [19] and compatibility with SHapley Additive exPlanations (SHAP) [20], which is a method to interpret machine learning model. Nested stratified 10-fold cross-validation was used to avoid overfitting of model performance. To reduce the high dimensionality of gene expression data, glmmSeq was used to select differentially expressed genes according to the criteria described above, using the repeated-measures model described in the preceding section rather than separate timepoint-specific analyses. Gene selection was performed within classifier training to prevent information leakage between training and testing datasets and to minimize overfitting. Feature (gene) selection and model performance were evaluated using nested cross-validation, with an outer 10-fold cross-validation loop for model evaluation and an inner 5-fold loop for hyperparameter optimization. Within each outer training fold, feature selection was performed independently, allowing the stability of selected genes to be assessed across resampled training subsets while preserving independence of the held-out test data. Hyperparameter tuning of the XGBoost model was carried out using scikit-optimize’s Bayes search CV [21] within the inner cross-validation loop using a search over predefined parameter ranges. The parameters tuned included the number of trees (n_estimators), maximum tree depth (max_depth), learning rate, row subsampling ratio (subsample), column subsampling ratio per tree (colsample_bytree), minimum loss reduction for node splitting (gamma) and L1 and L2 regularization parameters (reg_alpha and reg_lambda).

The search ranges were: n_estimators (100–2,000), max_depth (1–10), learning_rate (0.001–1.0), subsample (0.05–1.0), colsample_bytree (0.05–1.0), gamma (0–0.7), reg_alpha (0–1) and reg_lambda (0–1). The optimal parameter combination was selected based on the mean area under the receiver operating characteristic curve (ROC–AUC) across the inner cross-validation folds. Three models were explored: (i) baseline only data; (ii) 4-week only; and (iii) inputs from both time-points (baseline and 4-week). To benchmark predictive performance against clinical characteristic data, an additional clinical characteristics only XGBoost classifier was trained, with hyperparameters optimized using 10-fold cross validation. Following fitting of the model, the model was interpreted using SHAP values averaged over 1,000 initializations of gradient boosted trees to account for variability in this stochastic process. Finally, features with mean absolute SHAP values of at least 0.05 were considered the most important features and were used to train the final model, and the same set of features were used for functional enrichment analysis using Metascape [22].

### Cell deconvolution analysis

To assess whether prediction is driven by immune-cell composition rather than gene regulation within cells, cell-type proportions were estimated from RNA-seq data using CIBERSORTx [23] with the LM22 reference panel. Downstream transformation, statistical testing and multiple-testing correction were performed according to our previously published workflow [13].

## Results

Following data quality control 16 548 genes were available for analysis in 100 patients. Patients were either categorized as responders (*n* = 69) or non-responders (*n* = 31) following 6 months of MTX therapy. The baseline characteristics of the patients are presented in Table 1. Baseline characteristics were broadly comparable between responders and non-responders, with no statistically significant differences observed for the variables listed in Table 1, except for baseline DAS28-CRP.

**Table 1:** Baseline patient characteristics of early RA patients participating in the study.

Characteristic		Grouped by response			
		Overall	NR	R	*P*-value
*n*		100	31	69	
Sex, *n* (%)	Female	79 (79.0)	28 (35.4)	51 (64.6)	0.110
	Male	21 (21.0)	3 (14.3)	18 (85.7)	
Concurrent csDMARD usage, *n* (%)	No	77 (77.0)	22 (28.6)	55 (71.4)	0.482
	Yes	23 (23.0)	9 (39.1)	14 (60.9)	
Age, mean (SD)		59.9 (12.4)	58.3 (12.5)	60.6 (12.3)	0.392
DAS28-CRP, mean (SD)		4.7 (1.0)	4.4 (0.9)	4.8 (1.0)	0.026
HAQ score, mean (SD)		1.4 (0.7)	1.5 (0.8)	1.3 (0.7)	0.467
BMI, mean (SD)		29.4 (6.5)	30.5 (6.7)	28.9 (6.4)	0.291
Disease duration, median [Q1, Q3]		0.3 [0.2,1.3]	0.4 [0.2,2.4]	0.3 [0.2,1.1]	0.256
Smoking status, *n* (%)	Never smoked	51 (51.5)	17 (33.3)	34 (66.7)	0.633
	Current smoker	13 (13.1)	5 (38.5)	8 (61.5)	
	Ex-smoker	35 (35.4)	9 (25.7)	26 (74.3)	
CRP, median [Q1, Q3]		7.7 [3.4,21.1]	5.4 [2.6,9.1]	11.3 [4.6,28.4]	0.015

*P*-values were obtained using two-sample *t*-tests for continuous variables including age, DAS28-CRP and HAQ score; chi-squared tests for categorical variables including sex, concurrent csDMARD usage and smoking status; and the Kruskal–Wallis test for disease duration and CRP, given its non-normal distribution.

DAS28-CRP: disease activity score in 28 joints with C-reactive protein; csDMARD: conventional synthetic disease modifying anti-rheumatic drugs; HAQ Score: health assessment questionnaire score; BMI: body mass index; NR: non-responder; R: responder.

### Patient baseline characteristics: differential expression

Ninety-three genes were differentially expressed between responders and non-responders across both timepoints. Eighty-one of these were not statistically significant in the interaction term; that is, they were not dependent on time. Arachidonate 15-Lipoxygenase, Type B (*ALOX15B*) was the most upregulated gene in responders in the mixed effect model, which accounted for both timepoints, showing a fold change of 1.7. This was followed by Oleoyl-ACP Hydrolase (*OLAH*) and CUB and Sushi Multiple Domains 1 (*CSMD1*) with fold changes of 1.56 and 1.58, respectively (Table 2). Nebulette (*NEBL*), associated with actin cytoskeletal organization, was the most downregulated gene in responders, with a fold change of 0.52 (Table 2). Twelve differentially expressed genes were dependent on time (Figure S1). Expression of *OLAH*, *IGHV3-72* and *AMPH* was reduced in responders by 0.76, 0.76 and 0.68-fold over the 4 weeks, respectively (Table 2, Fig. 1). Enrichment analysis with Metascape revealed that the majority of response associated genes were enriched in adaptive immune response and fatty acid biosynthetic process (Figures S2–S4).

**Figure 1:**
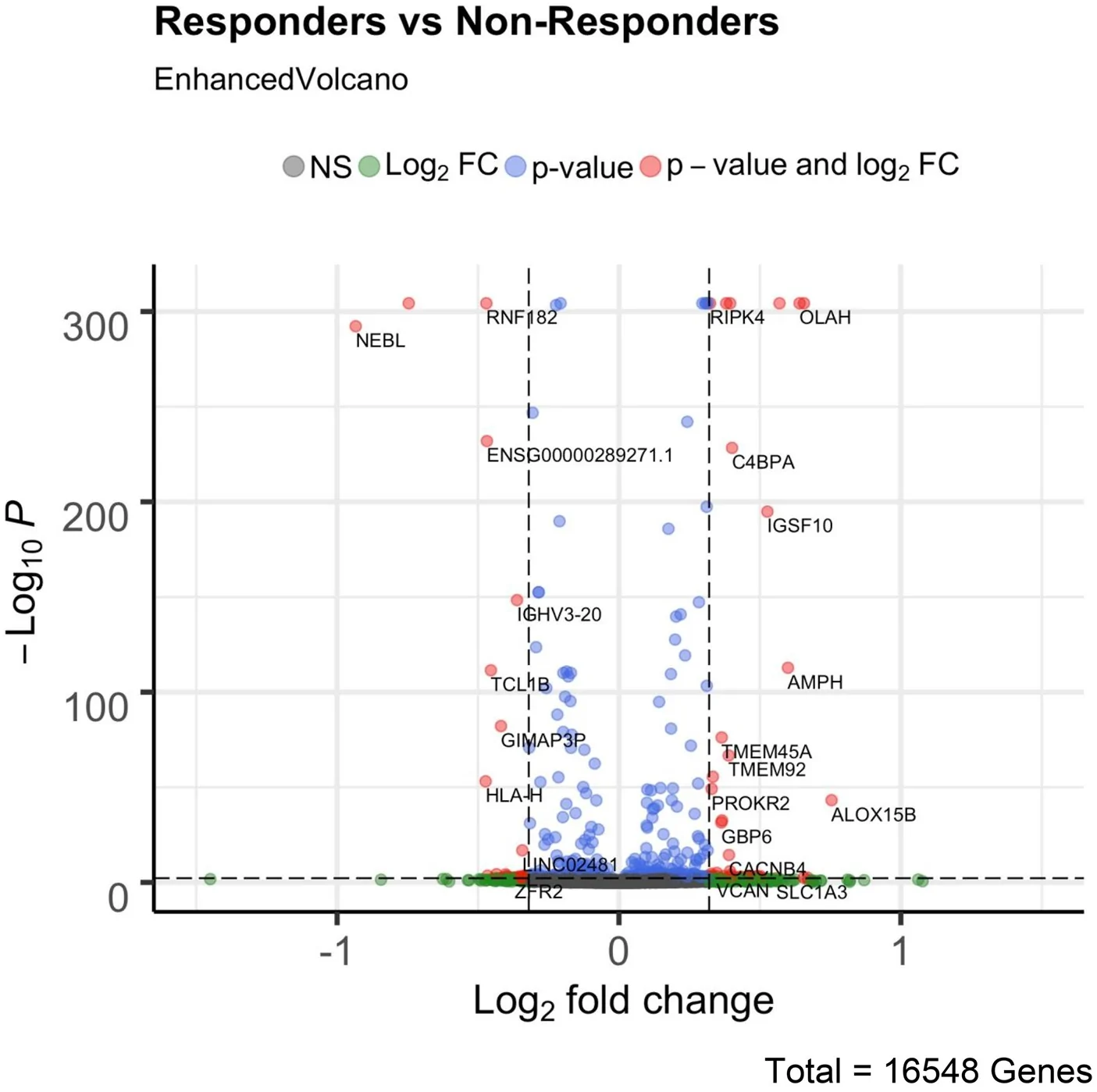
Volcano plot of glmmSeq model highlighting differentially expressed genes. Log_2_ fold change of more than zero represents genes upregulated in responders or downregulated in non-responders. Log_2_ fold change of less than zero represents genes downregulated in responders or upregulated in non-responders. Genes are colourized based on surpassing FDR below 0.05 and/or a fold-change exceeding 1.25

**Table 2:** List of differentially expressed genes from the glmmSeq analysis with the coefficient presented for the response outcome (Response), sampling timepoint (Timepoint), and interaction between response outcome and sampling timepoint (Interaction), the FDR-q is the FDR adjusted q-value. Brief relevant gene annotation are presented in the biological annotation column.

Gene symbol	Response	Response FDR-q	Timepoint	Timepoint FDR-q	Interaction	Interaction FDR-q	Biological annotation
*ALOX15B*	1.69 ± 1.04	8.93E-42	1.15 ± 1.16	1.00E + 00	0.74 ± 1.20	1.00E + 00	Involved in lipid metabolism and inflammation; implicated in inflammatory diseases and cancers [24].
*OLAH*	1.56 ± 1.01	9.10E-299	1.12 ± 1.01	7.44E-32	0.76 ± 1.01	4.75E-194	Participates in fatty acid biosynthesis; associated with rheumatoid arthritis [25].
*CSMD1*	1.58 ± 1.01	9.30E-299	0.99 ± 1.01	9.63E-01	1.07 ± 1.01	1.69E-07	Acts as a complement control protein; associated with tumour suppression and cancers [26].
*NEBL*	0.52 ± 1.02	3.60E-290	1.03 ± 1.02	8.28E-01	0.98 ± 1.02	1.00E + 00	Involved in cardiac and skeletal muscle integrity [27].
*PGF*	0.99 ± 1.27	5.35E-01	5.81 ± 1.27	2.04E-34	0.94 ± 1.34	1.00E + 00	Key regulator of angiogenesis; elevated in cancer [28].
*XIRP2*	1.27 ± 1.16	3.25E-01	1.65 ± 1.16	2.24E-03	0.80 ± 1.19	1.00E + 00	Maintains cardiac muscle structure; associated with arrhythmias and cardiomyopathies [29].
*IGLV1-36*	0.86 ± 1.02	1.98E-22	0.61 ± 1.02	3.80E-164	1.40 ± 1.02	1.08E-104	Encodes immunoglobulin light chain variable region.
*IGHV3-20*	0.78 ± 1.01	1.67E-146	0.64 ± 1.01	9.15E-299	1.24 ± 1.01	9.79E-104	Encodes immunoglobulin heavy chain variable region;
*CAV1*	0.92 ± 1.01	5.11E-21	0.75 ± 1.01	3.28E-250	1.27 ± 1.01	3.26E-181	Scaffolding protein regulating cell signalling; implicated in cancers and cardiovascular diseases [30].
*SDC1*	1.24 ± 1.00	9.70E-299	0.53 ± 1.00	9.10E-299	1.25 ± 1.00	9.63E-299	Cell adhesion protein; associated with cancer progression and wound healing [31].
*IGHV3-72*	1.48 ± 1.01	9.90E-299	1.07 ± 1.13	5.95E-02	0.74 ± 1.01	9.84E-299	Encodes immunoglobulin heavy chain variable region;
*AMPH*	1.52 ± 1.02	5.07E-111	1.21 ± 1.02	1.00E-03	0.68 ± 1.02	1.51E-91	Involved in endocytosis; autoantibodies linked to paraneoplastic neurological syndromes [32].

### Machine learning classifier of methotrexate response

A machine learning classifier for predicting treatment response outcome built using only clinical characteristics was first explored (not shown). Firstly, a basic clinical model including patient sex and baseline DAS28-CRP was developed and achieved a mean internal cross-validation AUC of 0.68 (± 0.13). A second, more complex model including all clinical information listed in Table 1 achieved similar performance with a mean internal cross-validation AUC of 0.72 (± 0.11). Transcriptomics model development retained patient sex and baseline DAS28-CRP to maintain a parsimonious model and reduce the risk of overfitting. The machine learning model that included gene expression and clinical information incorporating both timepoints performed less well than single timepoint models, with an AUC of ∼0.84. We therefore prioritized the models built with gene expression and clinical information that require less sampling. These models, stratified by individual timepoints, achieved an AUC of 0.89 (± 0.09) and 0.90 (± 0.08) for the baseline and 4-week classifier, respectively (Fig. 2). Both models also achieved good performance in other classification metrics (Table 3).

**Figure 2:**
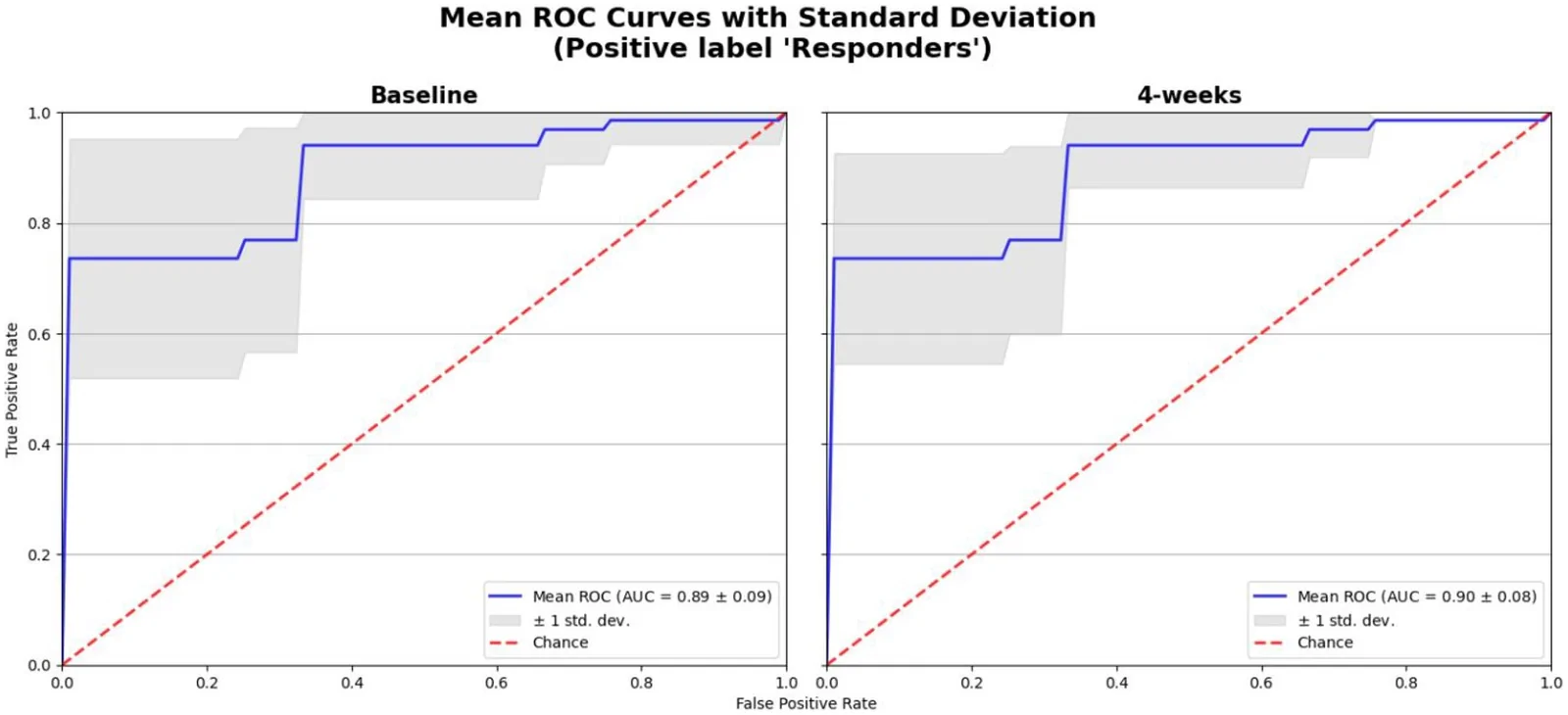
Receiver operating characteristic curve for baseline classifier (left) and 4-weeks classifier (right)

**Table 3:** Classification results for both classifier models. Performance metrics are reported as the mean ± standard deviation across the 10-fold cross-validation folds. AUC-PRS: area under the curve of precision recall curve. F1-Score is harmonic mean of precision and recall, which summarizes the classification performance. Accuracy is the proportion of correctly classified samples. Balanced accuracy, calculated as the mean of sensitivity and specificity, was additionally reported to account for class imbalance between responders and non-responders.

Metric	Baseline model	4-weeks model
AUC-PRS	0.93 ± 0.05	0.87 ± 0.08
F1-Score	0.80 ± 0.10	0.75 ± 0.10
Accuracy	0.73 ± 0.12	0.66 ± 0.13
Balanced accuracy	0.70 ± 0.12	0.61 ± 0.14

Using mean absolute SHAP values of 0.05 as cut-off, the baseline model had 33 features surpassing this threshold and the 4-weeks model had 54. One thousand initialization of gradient boosted trees were evaluated for SHAP values, and their standard deviations were computed and shown in Fig. 3; the standard deviations are larger than the mean for some features, particularly seen in the 4-week model. Among the important set of genes, six genes at baseline and 15 genes at week 4 have unknown functions, with annotations available only through their Ensembl IDs or as known long non-coding RNAs.

**Figure 3:**
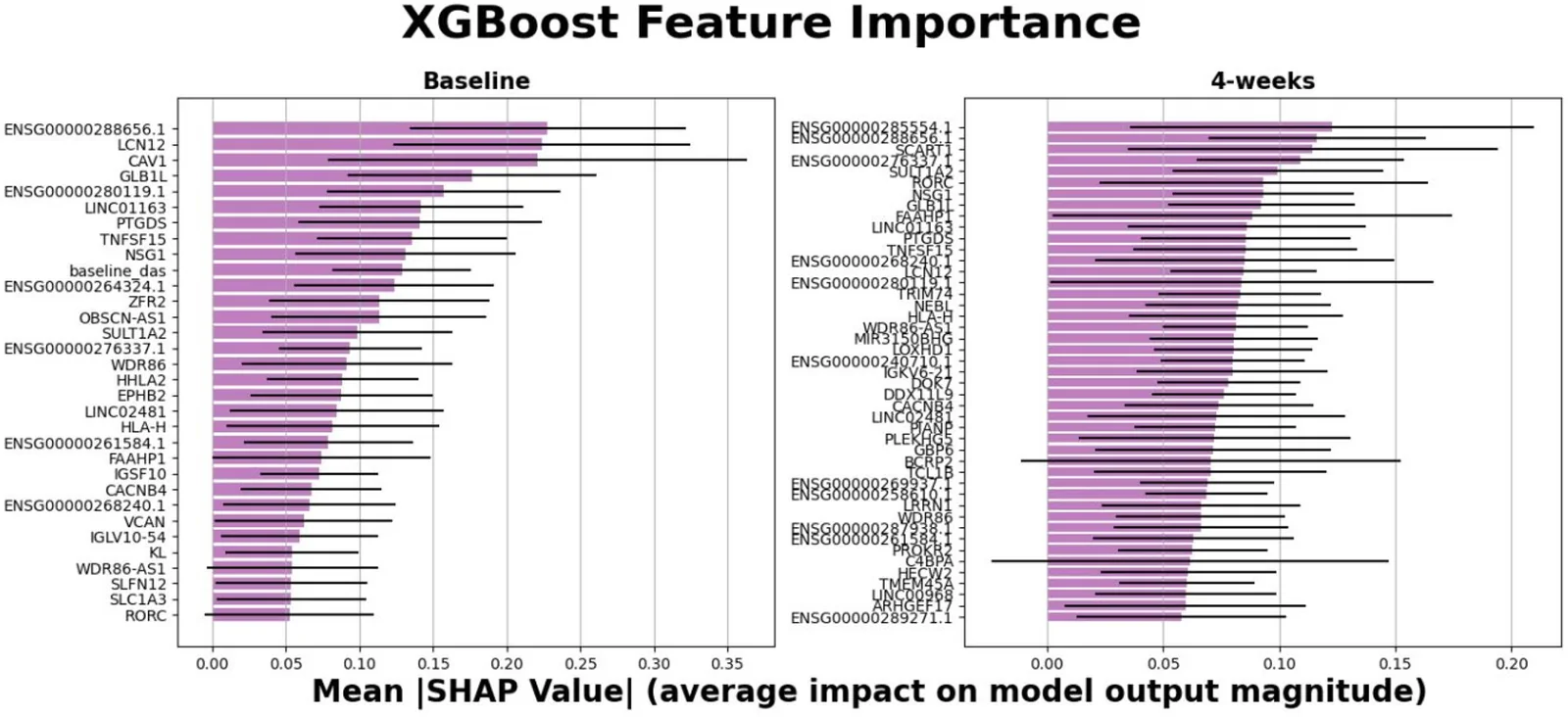
Feature importance for gradient boosted trees classifiers generated based on mean absolute SHAP values. Standard deviation of the SHAP values generated from 1000 initialization are represented on the bar plots as black lines. Left is the baseline classifier and right is the 4-weeks classifier

At baseline, the three most informative genes with known annotations are Lipocalin-12 (*LCN12*), Caveolin-1 (*CAV1*) and Galactosidase Beta 1 Like *(GLB1L)*. The first two genes demonstrated significant downregulation in responders, with *CAV1* downregulated by 0.92, *LCN12* by 0.79, but *GLB1L* was upregulated by 1.26. *CAV1* is a scaffolding protein that plays a critical role in membrane structure and signal transduction. It has been implicated in cancer, cardiovascular and pulmonary diseases due to its tumour suppressor functions and regulatory influence on cellular pathways [33]. *LCN12* belongs to the lipocalin family and is involved in the transport of hydrophobic molecules such as steroids and has been associated with inflammation and cancer [34]. *GLB1L* encodes a predicted β-galactosidase-like protein within the glycosyl hydrolase family, suggesting potential involvement in glycan processing pathways, although its precise biological role remains poorly characterized [35]. Other notable genes in the baseline model include: lipid transport protein prostaglandin D synthase (*PTGDS*), a potential prognostic marker in cancer that is associated with activation of immune cells [36]; TNF superfamily member 15 (*TNFSF15*), a member of tumour necrosis factor family that is inducible by TNF, is expressed on several immune cells and is abnormally expressed in inflammatory disease including RA [37]; and HERV-H LTR-Associating 2 (*HHLA2*) an immune checkpoint molecule that has immune inhibitory and stimulatory functions, depending on the type of receptor it binds to [38].

At week 4, the three most informative genes with known annotations were Scavenger Receptor Family Member Expressed on T Cells 1 *(SCART1)*, sulfotransferase family 1A member 2 (*SULT1A2*) and RAR-Related Orphan Receptor C *(RORC)*. *SCART1* and *RORC* exhibited a 21% downregulation in responders, while *SULT1A2* is upregulated by 32%. *SCART1* is a gene that likely functions as a co-receptor on αβ and γδ T cells, playing a role in immune system regulation; however, its specific functions and disease associations remain poorly characterized. *SULT1A2* encodes a cytosolic sulfotransferase belonging to the SULT1 family, which catalyses sulphf conjugation reactions in phase II metabolism; however, the precise biological role of SULT1A2 remains less well defined than that of better studied sulfotransferases [39]. *RORC* is a nuclear receptor that functions as a DNA-binding transcription factor essential for lymphoid organogenesis and thymopoiesis. Dysregulation of *RORC* has been associated with immunodeficiency and inflammatory diseases [40]. Both gene sets were statistically significantly enriched in immune system processes.

Estimated immune-cell proportions derived from CIBERSORTx were not significantly different between responders and non-responders, with all comparisons yielding unadjusted *P*-values >0.05.

## Discussion

In this study, we performed whole-blood RNA-sequencing at pre-treatment and following 4-weeks on treatment to develop a classifier of MTX response by 6 months and identify gene biomarkers in responders and non-responder patient groups, where responders are patients with EULAR treatment response classification of good and moderate. This grouping was selected to capture informative biomarker profiles prior to any improvement on treatment. Overall, genes found to be associated with response either through statistical modelling or machine learning model were enriched in adaptive immune responses. The machine learning models in this study achieved a higher AUC of ∼0.89 when individual timepoints were analysed, an improvement over previous blood-based studies using microarrays [8] or in smaller sample numbers [41].

Among the differentially expressed genes, several have established associations with RA. For instance, *ALOX15B* is involved in the metabolism of polyunsaturated fatty acids and has been implicated in inflammatory processes and is upregulated in RA patients [42]. *OLAH* and Versican (*VCAN*) are involved in fatty acid biosynthesis and cell adhesion in RA patients within bone marrow and synovial fibroblasts, respectively [25]. All three genes were upregulated in responders with expressions decreasing in the post treatment sample (Supplementary Fig. S5). Other genes that were upregulated in responders but decreased following treatment with methotrexate included *IGHV3-72* and *AMPH*.

The machine learning approach revealed that the baseline model was the most informative and stable, based on dispersion of SHAP values. The model trained on the 4-week timepoint resulted in a lower score on all the metrics as well as high standard deviation on the SHAP values, indicating potential overfitting and less generalizable results. The difference in genes identified between the statistical model and machine learning model were observed with high effect sizes or low *P*-value not translating to high mean absolute SHAP values (Supplementary Table S1). Despite this, the genes identified by both methods are implicated in various processes with relevance to RA including inflammation and atherogenesis: *ALOX15B* [43]; complement activation: *CSMD1* [44]; cell proliferation, migration and adhesion: *SDC1* [31] and proliferation and cytokine production in T cells: *HHLA2.* HHLA2 can either bind to the receptor KIR3DL3 or TMIGD2. TMIGD2 is expressed on various subsets of T cells and Natural Killer (NK) cells and interaction with HHLA2 [38] results in enhanced proliferation and cytokine production. Conversely, binding of HHLA2 to KIR3DL3 results in inhibition of T cells and NK cell pathways [38]. Given the dual role of HHLA2, the importance of checkpoint molecules in both cancer and inflammatory diseases [45], and the identification of *HHLA2* in both analyses presented here, this molecule deserves further investigation in independent experiments.

The identified genes also revealed a theme of apoptosis. For example, Klotho (*KL*) and *VCAN* were up-regulated in responders with 1.27-fold and 1.4-fold change, respectively, whilst *NSG1* was down-regulated in responders with 0.76-fold change. They were ranked 26, 28 and 9 based on baseline classifier’s mean absolute SHAP values. *NSG1* is a known mediator of p53 inducing apoptosis [46], while *KL* has been reported to promote apoptosis in cancer cells [47]; in contrast, *VCAN* has been shown to exert anti-apoptotic effects, particularly under oxidative stress conditions [48]. The involvement of these genes may indicate a possible link between MTX use and apoptotic processes, aligning with the drug’s known mechanisms of action [49]. However, experiments in enriched cell types that are relevant to RA are required to investigate this further.

The current study has several strengths and limitations that should be acknowledged. The RAMS study is observational and is vulnerable to biases and confounding that may only partially be controlled for during data analysis. Common confounding would include patients having different baseline DAS28, which were accounted for during analysis. Further, the use DAS28-CRP composite score is likely to capture information beyond inflammation or mechanisms that have a clear biological basis. Although CDAI would provide an alternative outcome measure that does not incorporate CRP, its use was not possible because of the high proportion of missing data for the physician global assessment component. The feature selection strategy employed may not have captured the full spectrum of gene interaction because the selection was carried using univariate analysis prior to machine learning modelling. While this approach reduced the dimensionality of gene-expression matrix and helped limit overfitting in the context of a modest sample size, it may have excluded genes with predictive value only in combination with other genes. Future studies with larger cohorts could compare alternative feature-selection strategies, including variance-based filtering, penalized regression and embedded feature selection methods. Strengths include the comprehensive RNA sequencing approach across multiple time points, allowing the change in expression profile following treatment to be observed. The machine learning pipeline employed robust internal validation coupled to the powerful gradient boosting tree algorithm that can incorporate weights into the model to account for class imbalance. Although we mitigated overfitting with the modest sample size by cross-validation, the lack of validation in an independent external cohort is a limitation that prevents assessment of the generalizability of the model.

## Conclusion

In conclusion, this study provides valuable insights into the genomics underpinnings of MTX response in RA, offering a basis for future research aimed at improving personalized treatment strategies by including blood-based biomarker profiling. Despite the limitations outlined above, the study’s novel approach and significant findings contribute to the growing body of knowledge in RA treatment and precision medicine, but findings require independent replication.

## Supplementary Material

keag560_Supplementary_Data

## Data Availability

Data available upon request. Sharing and will be done in line with University of Manchester recommended procedures and funding requirements. The ethical approval [North West 6 Central Manchester South Research Ethics Committee (COREC 04/Q1403/37)] allows sharing of data with other bona-fide researchers both in the UK and abroad.

## References

[1] Smolen JS , Landewé RBM, Bergstra SA et al EULAR recommendations for the management of rheumatoid arthritis with synthetic and biological disease-modifying antirheumatic drugs: 2022 update. Ann Rheum Dis 2023;82:3–18. 10.1136/ard-2022-223356 36357155

[2] Taylor PC , Woods M, Rycroft C et al Targeted literature review of current treatments and unmet need in moderate rheumatoid arthritis in the United Kingdom. Rheumatology (Oxford) 2021;60:4972–81. 10.1093/rheumatology/keab464 PMC8566217 34080612

[3] Salliot C , van der Heijde D. Long-term safety of methotrexate monotherapy in patients with rheumatoid arthritis: a systematic literature research. Ann Rheum Dis 2009;68:1100–4. 10.1136/ard.2008.093690 PMC2689525 19060002

[4] Smolen JS , Landewé RBM, Bijlsma JWJ et al EULAR recommendations for the management of rheumatoid arthritis with synthetic and biological disease-modifying antirheumatic drugs: 2019 update. Ann Rheum Dis 2020;79:685–99. 10.1136/annrheumdis-2019-216655 31969328

[5] Gwinnutt JM , Symmons DPM, MacGregor AJ et al Twenty-year outcome and association between early treatment and mortality and disability in an inception cohort of patients with rheumatoid arthritis: results from the Norfolk arthritis register. Arthritis Rheumatol 2017;69:1566–75. 10.1002/art.40090 PMC5600136 28425173

[6] Bluett J , Sergeant J, MacGregor AJ, Symmons D, Verstappen S. Predictors of oral methotrexate failure in patients with early inflammatory arthritis: a competing risks analysis. Rheumatology 2015;54:i71.

[7] Sergeant JC , Hyrich KL, Anderson J et al; RAMS Co-Investigators. Prediction of primary non-response to methotrexate therapy using demographic, clinical and psychosocial variables: results from the UK Rheumatoid Arthritis Medication Study (RAMS). Arthritis Res Ther 2018;20:147. 10.1186/s13075-018-1645-5 PMC6044018 30005689

[8] Plant D , Maciejewski M, Smith S et al; Maximising Therapeutic Utility in Rheumatoid Arthritis Consortium, the RAMS Study Group. Profiling of gene expression biomarkers as a classifier of methotrexate nonresponse in patients with rheumatoid arthritis. Arthritis Rheumatol 2019;71:678–84. 10.1002/art.40810 PMC9328381 30615300

[9] Luedders BA , Johnson TM, Sayles H et al Predictive ability, validity, and responsiveness of the multi-biomarker disease activity score in patients with rheumatoid arthritis initiating methotrexate. Semin Arthritis Rheum 2020;50:1058–63. 10.1016/j.semarthrit.2020.05.019 PMC7584754 32911284

[10] Gosselt HR , Muller IB, Jansen G et al Identification of metabolic biomarkers in relation to methotrexate response in early rheumatoid arthritis. JPM 2020;10:271. 10.3390/jpm10040271 PMC7768454 33321888

[11] Arnett FC , Edworthy SM, Bloch DA et al The American Rheumatism Association 1987 revised criteria for the classification of rheumatoid arthritis. Arthritis Rheum 1988;31:315–24. 10.1002/art.1780310302 3358796

[12] Fransen J , Van Riel P. The Disease Activity Score and the EULAR response criteria. Clin Exp Rheumatol 2005;23:S93–9. 16273792

[13] Yap CF , Nair N, Morgan AW et al Machine learning analysis of whole-blood transcriptomics data in rheumatoid arthritis patients treated with adalimumab identifies predictive biomarkers of response. Arthritis Rheumatol 2025;77:1663–72. 10.1002/art.43255 PMC12750119 40415615

[14] Dobin A , Davis CA, Schlesinger F et al STAR: ultrafast universal RNA-seq aligner. Bioinformatics 2013;29:15–21. 10.1093/bioinformatics/bts635 PMC3530905 23104886

[15] Li B , Dewey CN. RSEM: accurate transcript quantification from RNA-Seq data with or without a reference genome. BMC Bioinformatics 2011;12:323. 10.1186/1471-2105-12-323 PMC3163565 21816040

[16] Myles Lewis KG , Sciacca E, Cubuk C, Surace A. glmmSeq: general linear mixed models for gene-level differential expression. R package version 0.5.4. 2021. https://github.com/myles-lewis/glmmSeq (29 November 2022, date last accessed).

[17] Love MI , Huber W, Anders S. Moderated estimation of fold change and dispersion for RNA-seq data with DESeq2. Genome Biol 2014;15:550. 10.1186/s13059-014-0550-8 PMC4302049 25516281

[18] Chen T , Guestrin C. XGBoost: a scalable tree boosting system. In: Proceedings of the 22nd ACM SIGKDD International Conference on Knowledge Discovery and Data Mining. New York: ACM, 2016:785–94.

[19] Grinsztajn L , Oyallon E, Varoquaux G. Why do tree-based models still outperform deep learning on tabular data? In: NIPS'22: Proceedings of the 36th International Conference on Neural Processing Systems. 2022;37:507–20.

[20] Lundberg SM , Lee S-I. A unified approach to interpreting model predictions. In: Proceedings of the 31st International Conference on Neural Information Processing Systems, 2017. pp. 4768–77. Curran Associates Inc., Long Beach, CA, USA.

[21] Tim H , MechCoder Gilles L, Iaroslav S et al scikit-optimize/scikit-optimize: v0.5.2. Zenodo, 2018. https://zenodo.org/records/1207017 (22 December 2023, date last accessed).

[22] Zhou Y , Zhou B, Pache L et al Metascape provides a biologist-oriented resource for the analysis of systems-level datasets. Nat Commun 2019;10:1523. 10.1038/s41467-019-09234-6 PMC6447622 30944313

[23] Newman AM , Steen CB, Liu CL et al Determining cell type abundance and expression from bulk tissues with digital cytometry. Nat Biotechnol 2019;37:773–82. 10.1038/s41587-019-0114-2 PMC6610714 31061481

[24] Tian R , Zuo X, Jaoude J et al ALOX15 as a suppressor of inflammation and cancer: lost in the link. Prostaglandins Other Lipid Mediat 2017;132:77–83. 10.1016/j.prostaglandins.2017.01.002 PMC5509529 28089732

[25] Nakamura N , Shimaoka Y, Tougan T et al Isolation and expression profiling of genes upregulated in bone marrow-derived mononuclear cells of rheumatoid arthritis patients. DNA Res 2006;13:169–83. 10.1093/dnares/dsl006 17082220

[26] Kamal M , Shaaban AM, Zhang L et al Loss of CSMD1 expression is associated with high tumour grade and poor survival in invasive ductal breast carcinoma. Breast Cancer Res Treat 2010;121:555–63. 10.1007/s10549-009-0500-4 19669408

[27] Mastrototaro G , Liang X, Li X et al Nebulette knockout mice have normal cardiac function, but show Z-line widening and up-regulation of cardiac stress markers. Cardiovasc Res 2015;107:216–25. 10.1093/cvr/cvv156 PMC4565986 25987543

[28] Escudero-Esparza A , Martin TA, Davies ML, Jiang WG. PGF isoforms, PLGF-1 and PGF-2, in colorectal cancer and the prognostic significance. Cancer Genomics Proteomics 2009;6:239–46. 19657001

[29] Wang Q , Lin JL, Erives AJ, Lin CI, Lin JJ. New insights into the roles of Xin repeat-containing proteins in cardiac development, function, and disease. Int Rev Cell Mol Biol 2014;310:89–128. 10.1016/B978-0-12-800180-6.00003-7 PMC4857591 24725425

[30] Nwosu ZC , Ebert MP, Dooley S, Meyer C. Caveolin-1 in the regulation of cell metabolism: a cancer perspective. Mol Cancer 2016;15:71. 10.1186/s12943-016-0558-7 PMC5112640 27852311

[31] Akl MR , Nagpal P, Ayoub NM et al Molecular and clinical profiles of syndecan-1 in solid and hematological cancer for prognosis and precision medicine. Oncotarget 2015;6:28693–715. 10.18632/oncotarget.4981 PMC4745686 26293675

[32] Nakashima K , Fujii Y, Sato M et al A case of non-small cell lung cancer presenting anti-amphiphysin antibody-positive paraneoplastic neurological syndrome. Respir Med Case Rep 2021;34:101525. 10.1016/j.rmcr.2021.101525 PMC8512629 34692399

[33] Dalton CM , Schlegel C, Hunter CJ. Caveolin-1: a review of intracellular functions, tissue-specific roles, and epithelial tight junction regulation. Biology (Basel) 2023;12:1402. 10.3390/biology12111402 PMC10669080 37998001

[34] Charkoftaki G , Wang Y, McAndrews M et al Update on the human and mouse lipocalin (LCN) gene family, including evidence the mouse Mup cluster is result of an “evolutionary bloom”. Hum Genomics 2019;13:11. 10.1186/s40246-019-0191-9 PMC6381713 30782214

[35] Gene [Internet]. Bethesda (MD): National Library of Medicine (US), National Center for Biotechnology Information; 2004 – . Gene ID 79411, GLB1L (galactosidase beta-1-like). https://www.ncbi.nlm.nih.gov/gene/79411 (11 December 2024, date last accessed).

[36] Li J , Qu Z, Zhu D et al Pan cancer research reveals the role of PTGDS in tumor suppression and immune regulation. NPJ Precis Oncol 2025;9:319. 10.1038/s41698-025-01097-z PMC12484698 41028158

[37] Xu W-D , Li R, Huang A-F. Role of TL1A in inflammatory autoimmune diseases: a comprehensive review. Front Immunol 2022;13:891328. 10.3389/fimmu.2022.891328 PMC9329929 35911746

[38] Li Y , Lv C, Yu Y et al KIR3DL3-HHLA2 and TMIGD2-HHLA2 pathways: the dual role of HHLA2 in immune responses and its potential therapeutic approach for cancer immunotherapy. J Adv Res 2023;47:137–50. 10.1016/j.jare.2022.07.013 PMC10173190 35933091

[39] Gamage N , Barnett A, Hempel N et al Human sulfotransferases and their role in chemical metabolism. Toxicol Sci 2006;90:5–22. 10.1093/toxsci/kfj061 16322073

[40] Torres-Huerta A , Ruley-Haase K, Reed T et al Retinoid orphan receptor gamma t (rorγt) promotes inflammatory eosinophilia but is dispensable for innate immune-mediated colitis. PLoS One 2024;19:e0300892. 10.1371/journal.pone.0300892 PMC10956760 38512959

[41] Brynedal B , Yoosuf N, Ulfarsdottir TB et al Molecular signature of methotrexate response among rheumatoid arthritis patients. Front Med (Lausanne) 2023;10:1146353. 10.3389/fmed.2023.1146353 PMC10084884 37051216

[42] Mao X , Li W, Chen W et al Exploring and characterizing a novel combination of paeoniflorin and talatizidine for the treatment of rheumatoid arthritis. Pharmacol Res 2020;153:104658. 10.1016/j.phrs.2020.104658 32001347

[43] Magnusson LU , Lundqvist A, Karlsson MN et al Arachidonate 15-lipoxygenase type B knockdown leads to reduced lipid accumulation and inflammation in atherosclerosis. PLoS One 2012;7:e43142. 10.1371/journal.pone.0043142 PMC3422220 22912809

[44] Kraus DM , Elliott GS, Chute H et al CSMD1 is a novel multiple domain complement-regulatory protein highly expressed in the central nervous system and epithelial tissues. J Immunol 2006;176:4419–30. 10.4049/jimmunol.176.7.4419 16547280

[45] Spiliopoulou A , Iakovliev A, Plant D et al Genome-wide aggregated trans effects analysis identifies genes encoding immune checkpoints as core genes for rheumatoid arthritis. Arthritis Rheumatol 2025;77:817–26. 10.1002/art.43125 PMC12209750 39887658

[46] Kudoh T , Kimura J, Lu ZG, Miki Y, Yoshida K. D4S234E, a novel p53-responsive gene, induces apoptosis in response to DNA damage. Exp Cell Res 2010;316:2849–58. 10.1016/j.yexcr.2010.06.025 20599942

[47] Chen B , Wang X, Zhao W, Wu J. Klotho inhibits growth and promotes apoptosis in human lung cancer cell line A549. J Exp Clin Cancer Res 2010;29:99. 10.1186/1756-9966-29-99 PMC2912837 20642846

[48] Wu Y , Wu J, Lee DY et al Versican protects cells from oxidative stress-induced apoptosis. Matrix Biol 2005;24:3–13. 10.1016/j.matbio.2004.11.007 15748997

[49] Cronstein BN , Aune TM. Methotrexate and its mechanisms of action in inflammatory arthritis. Nat Rev Rheumatol 2020;16:145–54. 10.1038/s41584-020-0373-9 32066940

