# Supplementary material for: Identifying biomarkers for methotrexate efficacy in rheumatoid arthritis: a machine learning approach to whole-blood transcriptomic analysis
Source: Rheumatology (Oxford). 2026 Oct 8;65(11):keag560. doi: 10.1093/rheumatology/keag560 (PMC13743429; doi:10.1093/rheumatology/keag560)
Supplement: Supplementary file 1 [file keag560_supplementary_data.docx]

Supplementary Document for Identifying Biomarkers for Methotrexate Efficacy in Rheumatoid Arthritis: A Machine Learning Approach to Whole-Blood Transcriptomic Analysis

# Supplementary Results

Figure S1: Volcano plot of glmmSeq model of the summary statistics on interaction term between response outcome and time.

Figure S2: Metascape enrichment results for genes statistically significant for response outcome in negative binomial mixed effects model

Figure S3: Metascape enrichment results for genes statistically significant for sampling timepoint in negative binomial mixed effects model

Figure S4: Metascape enrichment results for genes statistically significant for interaction term between response outcome and sampling timepoint in negative binomial mixed effects model

Figure S5: Volcano plot of glmmSeq model of the summary statistics on fold-change of gene expression over time.

| **Genes** | **Response** | **Response FDR-q** | **Interaction** | **Interaction FDR-q** | **Baseline Mean absolute SHAP value** | **Baseline SHAP rank** | **4-weeks Mean absolute SHAP value** | **4-weeks SHAP rank** |
| --- | --- | --- | --- | --- | --- | --- | --- | --- |
| AMPH | 1.51 | 5.1E-111 | 0.66 | 1.51E-91 | 0.013 | 90 | 0.051 | 43 |
| NEBL | 0.52 | 3.6E-290 | 0.98 | 0.999971 | 0.030 | 58 | 0.074 | 20 |
| CAV1 | 0.92 | 5.11E-21 | 1.27 | 3.3E-181 | 0.28 | 2 | 0.035 | 69 |
| OLAH | 1.56 | 9.10E-299 | 0.76 | 4.7E-194 | 0.015 | 87 | 0.032 | 75 |
| ALOX15B | 1.69 | 8.93E-42 | 0.74 | 0.999971 | 0.016 | 82 | 0.030 | 80 |
| CSMD1 | 1.58 | 9.30E-299 | 1.07 | 1.69E-07 | 0.029 | 61 | 0.049 | 46 |
| IGLV1-36 | 0.86 | 1.98E-22 | 1.40 | 1.1E-104 | 0.033 | 53 | 0.030 | 81 |
| IGHV3-20 | 0.78 | 1.7E-146 | 1.24 | 9.8E-104 | 0.040 | 40 | 0.067 | 30 |
| IGHV3-72 | 1.48 | 9.90E-299 | 0.74 | 9.84E-299 | 0.017 | 76 | 0.021 | 91 |

Table S1: Comparison of differentially expressed genes and genes identified by the classifier models. The table includes genes that overlapped between the top differentially expressed genes identified using glmmSeq and the genes included in the classifier models. Response denotes the estimated coefficient for treatment response status, while Interaction denotes the estimated coefficient for the interaction between response status and sampling timepoint. Response FDR-q and Interaction FDR-q are the corresponding false discovery rate-adjusted q-values. Baseline mean absolute SHAP value and 4-weeks mean absolute SHAP value represent the average absolute contribution of each gene to the classifier predictions at baseline and four weeks, respectively. Baseline SHAP rank and 4-weeks SHAP rank indicate the relative importance of each gene within the corresponding classifier model, with a lower rank indicating greater predictive importance.
